# How is goal setting used in interventions for chronic disease prevention and management in sub-Saharan Africa? A systematic review and narrative synthesis

**DOI:** 10.1080/16549716.2025.2608423

**Published:** 2026-01-12

**Authors:** Cathryn Pinto, Matthew Burgess, Naomi Levitt, Peter Delobelle, Nuala McGrath

**Affiliations:** a School of Primary Care, Population Sciences, and Medical Education, Faculty of Medicine, University of Southampton, Southampton, UK; b Chronic Disease Initiative for Africa (CDIA), Department of Medicine, University of Cape Town, Cape Town, South Africa; c Department of Public Health, Vrije Universiteit Brussels, Brussel, Belgium; d Department of Social Statistics and Demography, Faculty of Social Sciences, University of Southampton, Southampton, UK; e Africa Health Research Institute, KwaZulu-Natal, South Africa; f School of Nursing & Public Health, University of KwaZulu-Natal, Durban, South Africa

**Keywords:** goals, non-communicable diseases, behaviour change, self-management, African

## Abstract

Non-communicable diseases are increasingly prevalent in sub-Saharan Africa, and goal setting is often used to promote healthy self-management behaviours. In this review, we aimed to synthesise literature around how goal setting is used, for application in future interventions in the region. A systematic search was conducted in six databases and results screened for eligibility. Study characteristics, intervention details, goal setting components, feedback from participants and facilitators were extracted. Data were analysed using narrative synthesis and thematic analysis. The Mixed Methods Appraisal Tool was used to assess study quality. We included 24 publications describing 18 unique interventions. Included studies were of high to moderate methodological quality. Goal setting intervention components were informed by a variety of frameworks and involved a range of tasks. Interventions were often facilitator-led; many were conducted in group settings. Participants reported goal setting as useful for putting self-management into practice but encountered challenges related to language and literacy levels. Adequate detail on goal setting intervention components was not always present. Through this review, we provide a comprehensive picture of the variability of goal setting approaches in chronic disease prevention and management in sub-Saharan Africa and recommend more standardised use and reporting of goal setting intervention components.

## Paper Context

- **Main findings:** Goal setting was used in 18 interventions for chronic disease prevention and management in sub-Saharan Africa. Participants and facilitators found goal setting useful for translating self-management advice into practice, however challenges included delivering structured goal setting activities, language barriers and literacy levels.
- **Added knowledge:** This review provides a comprehensive picture of the different ways that goal setting has been used for chronic disease prevention and management in sub-Saharan Africa and highlights participants’ and intervention facilitators’ experiences, barriers and facilitators to using goal setting.
- **Global health impact for policy and action:** The findings and recommendations can be used to design interventions that make better use of goal setting techniques, adapt them to the sub-Saharan African context and reporting guidelines for interventions that contain goal setting components have been provided.

## Background

Countries in sub-Saharan Africa (SSA) face an increasing prevalence and burden from non-communicable diseases (NCDs) [1], which constitute the second highest cause of death in the region [2]. The most common NCDs include cardiovascular diseases, diabetes, cancer and chronic respiratory diseases [3,4]. Recent systematic reviews have highlighted the increased prevalence and burden of type-2 diabetes, obesity, and hypertension in the region [5–7]. This, coupled with a shortage of healthcare workers and limited access to care, has made it important to encourage public health strategies to prevent disease onset and encourage management of NCDs through behavioural changes in the community [8].

NCD prevention and management often requires people to adopt behavioural and lifestyle changes, including monitoring symptoms, medication adherence, adoption of healthy behaviours such as physical activity or a combination of different behaviours. People living in low- and middle-income countries, including SSA, however may find it difficult to integrate lifestyle advice in their daily lives due to socio-economic and cultural determinants, such as poverty, cultural beliefs and the impact of the physical and food environment [9,10]. Some evidence suggests that there may be specific barriers to engagement with self-management behaviours in SSA. A review of self-management interventions in diabetes in SSA up to 2016 found that self-management practices were poor and adherence to self-care or risk-reduction behaviour was sub-optimal [10]. Similarly with HIV which can be a lifelong condition, a study using a motivational interviewing intervention to reduce the risk of onward HIV transmission in South Africa in 2008 reported low intervention engagement from adults living with HIV and implementation challenges for facilitators in setting action plans [11].

Goal setting – a process by which individuals identify a behavioural goal or aim that they would like to achieve in order to improve a health outcome – is a common strategy used in health behaviour change interventions for chronic disease prevention and management. Chronic disease prevention and management often require complex and multi-faceted treatment regimens or lifestyle changes, and goal setting can be used to motivate behaviour change and make these changes seem more attainable. In high income countries, goal setting has been found to improve intervention engagement, treatment outcomes, patient empowerment and activation [12–14]. Evidence from systematic reviews in diabetes self-management and physical activity promotion also show that goal setting is a key and effective component in fostering healthy behaviours and improving health outcomes [15,16]. However, there is considerable variability and limited evidence around the best ways to implement goal setting [17,18]. The way goals are framed and the individual’s circumstances and capability to achieve their goals are vital to using goal setting successfully. The socio-economic and cultural context in SSA is very different to high-income countries, and goal-setting techniques might need to be used differently in SSA to be successful at improving health outcomes.

Culturally sensitive interventions are important for improving engagement with health behaviours [19,20]. In SSA, cultural and environmental barriers exist in relation to self-care and self-management of chronic diseases [21,22]. For example, Alaofe and colleagues in Benin found that adults with Type 2 diabetes were not accustomed to self-directed blood glucose monitoring, which affected their adherence to treatment guidelines [21]. A study in rural Malawi identified several barriers to self-management across a number of NCDs, including poverty, availability of social support and disease stigma [23]. In regard to goal setting, a study in South Africa noted challenges with individual goal setting with respect to nutritional goals, which they hypothesised could have been due to low literacy levels among study participants [24].

Research conducted by our team on a couples-focused intervention to improve Type-2 diabetes self-management among those receiving care in public clinics indicated that participants struggled with SMARTER (Specific, Measurable, Achievable, Relevant, Time-bound, Evaluated, Reviewed) goals. Intervention facilitators also experienced challenges in presenting goal setting to the group vs one-to-one dialogues with individual partners and couples [25,26]. Even when using a simplified handout to introduce the SMARTER tool and presenting worked examples of setting a goal to the group, one-to-one facilitator support with couples was required, which made the activity difficult to conduct in a group setting.

A gap exists in our understanding of how goal setting is implemented and adapted to support preventative and NCD self-management interventions in SSA. The aim of this review was to synthesise existing literature on goal setting for chronic disease prevention and self-management and identify ways to use goal setting as a behaviour change strategy in this region. Our objectives are to describe the goal setting strategies used in SSA for chronic disease prevention and management and to explore contextual adaptations, understand views and experiences of study participants and facilitators, to gain insights to improve implementation of and engagement with goal setting. Understanding how to adapt goal setting techniques to the needs and preferences of people in SSA will enable the development of more effective interventions and programmes for chronic disease prevention and management.

## Methods

This was a systematic review with a narrative synthesis to comprehensively gather evidence on how goal setting is used in SSA and use the findings to inform gaps in knowledge and practice in order to use goal setting more effectively. The Preferred Reporting Items for Systematic Reviews and Meta-Analyses (PRISMA) statement was followed [27] and can be found in the supplementary files. The review protocol was pre-registered on PROSPERO and published as open access (CRD42024523544).

### Search strategy

A systematic search was carried out across six scientific databases, selected to cover international and regional studies conducted on both small and large scales: MEDLINE, PsycINFO, CINAHL Plus, Web of Science, Global Index Medicus and SCIELO. The search strategy and terms were developed by CP, MB and NM, in liaison with a librarian and informed by other goal setting reviews [15,28]. The search terms were piloted to ensure that all relevant studies were included and combined terms relating to goal-setting and self-management, chronic diseases or health promotion, interventions or programmes, and location in SSA countries, modified for each database and chosen to strike a balance between sensitivity and specificity of results based on pilot searches (see supplementary file 1). We also searched grey literature (ProQuest) and hand-searched the references of included studies at the final stage. No start date limits were applied, the end date was April 2024, and the search was limited to studies published in English. The final search results were imported to EndNote and duplicates removed.

### Screening and selection

**Figure 1.**
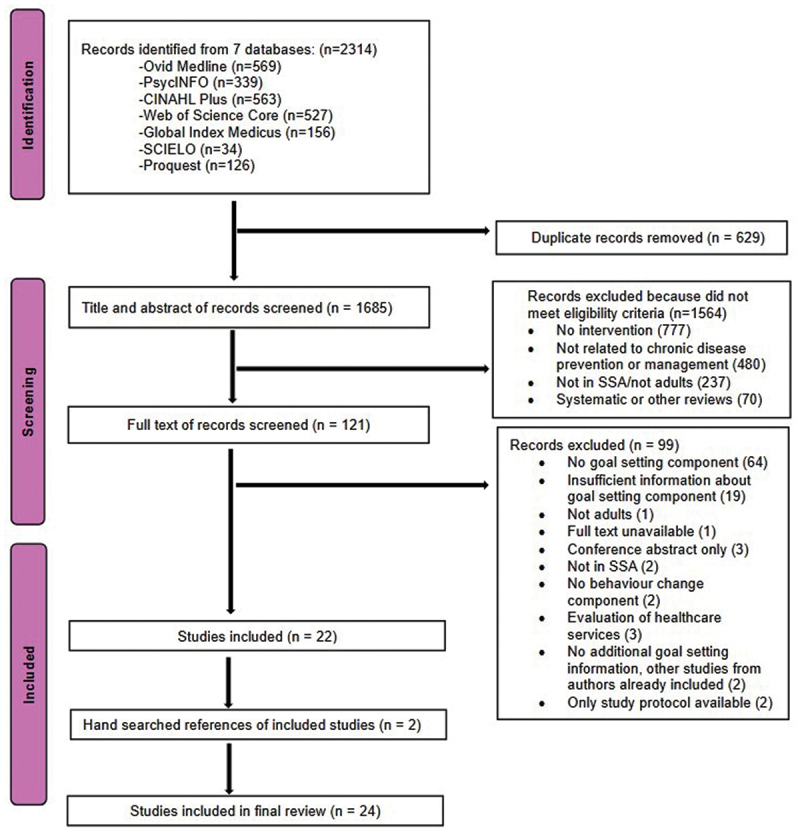
PRISMA diagram.

Studies describing interventions aiming to promote behaviour change to enhance chronic disease prevention for those who were at risk or to promote self-management and treatment adherence were selected for inclusion. Studies were included if they had a goal setting component and were carried out in SSA (Table 1). Chronic diseases were defined as diseases lasting for at least 3 months and which included an element of behaviour change as part of their treatment. HIV was included under the scope of chronic diseases if the study and intervention targeted people living with HIV and behaviour change to improve treatment adherence or to reduce the risk of onward transmission of HIV. Studies with qualitative, quantitative, and mixed-methods study designs, as well as intervention development papers and both observational and experimental study designs were included. Results that did not meet the inclusion criteria were excluded at the title and abstract stage. Studies were included for full-text screening when it was unclear from the title and abstract if the intervention had a goal setting component. Full texts were screened using the same criteria, and reasons for exclusion were recorded (Figure 1). In cases where information on the goal-setting intervention component was lacking in the full-texts, for example if goal setting was mentioned but there were no further details on how goal setting was implemented within the intervention, study authors were emailed for more information and additional papers considered for inclusion. Screening and selection were carried out by two authors (MB and CP) and decisions reviewed and resolved through discussion and consensus. In cases where consensus could not be reached, a third author (NM) was included in further discussions to reach consensus.

**Table 1.** Inclusion and exclusion criteria of selected studies.

Criteria	Inclusion	Exclusion
Population	Adults living in sub-Saharan African countries or a combination of SSA and non-SSA countries where the studies have disaggregated information for SSA Intervention facilitators who deliver interventions to support people with chronic disease self-management or prevention in SSA	Children or adolescents Adults not living in SSA
Intervention	Chronic disease management or prevention interventions that target behaviour change. These could be self-management, educational or supportive interventions or a combination. Interventions with a goal setting component.	Interventions that do not promote any behaviour change. Interventions with no goal setting component.
Comparator	Not applicable	
Outcomes	Behaviour change outcomes that centred around disease management or prevention or risk reduction (e.g. self-monitoring behaviours or medication adherence, or engaging in behaviours such as physical activity, eating healthily).	Outcomes not focused on changing behaviour.
Study design	Qualitative, quantitative and mixed methods studies. Studies describing intervention development processes and protocols provided some feedback or data from intervention target users was available.	Systematic reviews
Language	English	Languages other than, or translated into English

### Data extraction

The following information was extracted from each paper: study characteristics (authors, year, aim, study design, disease/health behaviour focus), participants (country, eligibility criteria, sample size), intervention (key features, intervention components and delivery method), goal setting component (approach used, types of goal setting tasks, delivery method and support, any cultural adaptations), and feedback from participants or those delivering the interventions specific to the goal setting intervention component. We also recorded the level of detail reported on goal setting for each study and based on our observations made recommendations for reporting goal setting intervention components for future studies.

### Quality appraisal

The Mixed Methods Appraisal Tool (MMAT) was used to assess the quality of included studies [29], because of its applicability across different study designs. The quality appraisal was conducted by two independent reviewers (CP and MB) and any disagreements resolved through consensus, resulting in allocation of an agreed quality rating.

### Data synthesis and analysis

A narrative synthesis following the framework of Popay et al. was used to synthesise the evidence from studies with heterogeneous intervention types and study results [30]. Characteristics and key findings from the included studies were summarised. Studies were grouped based on similarities in intervention and goal setting components, and the findings were tabulated, summarised, and presented as a table. Studies that contained data on experience and engagement with the intervention goal setting component were submitted to a separate data extraction and thematic analysis. These data were inductively coded; codes were compared across interventions and common patterns identified. At this stage initial themes were developed by identifying factors or elements that explained the similarities and differences between studies. The themes were refined and validated by using visual mapping and linking them to the study characteristics and key findings from the narrative synthesis to explore if they were related to specific health conditions, types of interventions or goal setting practices. Findings and interpretations were discussed within the research team, critically reflected upon, and further refined whilst writing up the results for publication. Using both a narrative synthesis and thematic analysis enabled us to both describe the range of goal setting interventions and develop analytic insights about participant engagement with goal setting to inform best practice.

### Ethics statement

This study is a systematic review and does not involve the recruitment of human participants but the analysis of data that has already been published. Therefore, the ethics statement does not apply to this study.

## Results

About 2314 records from six databases were identified. After removing duplicates, 1685 studies were assessed for eligibility of which 24 studies were included in the review. Figure 1 shows the screening and selection process and outcome at each stage. At the full-text screening stage, 19 publications had insufficient information on the goal setting component. Despite the corresponding authors of four of the 19 publications sharing additional publications that were linked to their primary publications, these four publications were excluded because they either did not provide sufficient information about goal setting (two studies) or described other interventions that they drew on but this information did not clearly correspond to the specific publications in our review (two studies). Two more full-text publications were included after hand-searching references of the included full-text publications.

### Study characteristics

The 24 publications in this review describe 18 unique interventions; some interventions were reported across more than one publication. Some papers referred to the same intervention but reported on different stages of intervention development or evaluation [24,31–39]. The studies were from seven different countries in SSA; half were in South Africa [24,31,37–46], four in Ghana [32,33,47,48], three in Ethiopia [22,35,36], three in Nigeria [49–51], two in Uganda [31,39], one in Mali [52], and one in Malawi [53]. Most (58%) of the studies targeted diabetes prevention [31,39,40,52] or management [24,34–38,46,47,49,50]; the remaining 42% were interventions for other conditions, including self-management of hypertension, chronic pain, and HIV [42–45,48,51,53] mental health [32,33] and women’s pre-conception health for those at risk of developing NCDs [41].

A large proportion (15/18) of the included interventions were facilitator-led [24,31–41,43–46,48,50–53], and many (11/18) were conducted in group settings [24,31–40,42–46,52]. Facilitators were either healthcare professionals (9/18 interventions) [22,24,32,33,35–38,44–46,48,50,51], community health workers or certified counsellors (3/18 interventions) [40,52,53], peer facilitators (2/18 interventions) [41,43], or a combination of peer facilitators and community health workers (1/18 interventions) [31,39]. Table 2 shows the detailed characteristics of included studies.

**Table 2.** Included study characteristics.

Author	Study aim	Location	Study design	Participants*	Focus area	Intervention description*
Absetz et al. [31] Van Olmen et al. [39]	A. To understand the needs and capacities of each SMART2D site while leveraging core strategies with key functions in a collaborative, phased process promoting cross-learning. B. To understand the implementation process and interaction with context in order to understand the results.	South Africa, Uganda	A. Intervention development description reporting findings from qualitative studies Qualitative study – process evaluation	Adults at increased risk of Type 2 diabetes (N in Uganda = 268; N in South Africa = 285)	Type 2 diabetes	SMART2D – Community support to support self-management through a peer group programme and care companions. Peer group leaders took part in a 2-day training workshop. Care companions included friends and family in Uganda and community health workers in South Africa. There were six self-management topics to guide peer support sessions.
Appiah et al. [32]; Appiah et al. [33];	A. To describe the development of the Inspired Life Programme designed to promote positive mental health and reduce depression. B. To explore participants’ experiences of, perceived benefits of, and recommendations to improve the Inspired Life Programme to promote positive mental health and reduce depression.	Ghana	A. Intervention development description with integrated qualitative components B. Qualitative study with post -intervention interviews about intervention experience	Participants from two rural, poor communities in Ghana (N for community consultation stage = 12; 6 men and 6 women)	Promoting positive mental health	10-session, multi-component positive psychology intervention to be delivered face-to-face by a trained facilitator (psychology graduates) in a group setting over two hours per week. A trained psychologist provided one week of intensive training on the intervention and group facilitation skills to the intervention facilitators.
Asante et al. [47]	To evaluate the feasibility and effectiveness of a nurse-led mobile phone call intervention on glycaemic management and adherence to self-management practices after 12 weeks among adults living with type 2 diabetes	Ghana	Pilot RCT	Adults living with Type 2 diabetes (*N* = 60)	Type 2 diabetes	Nurse-led phone calls on diabetes self-management for 12 weeks, each call lasted for up to 15 minutes. These calls reinforced the guidelines on self-management of diabetes according to the ‘Living with Diabetes’ book and included information on diet, exercise, medication, self-monitoring of blood glucose and foot care.
Catley et al. [40]	To evaluate the efficacy of ‘Lifestyle Africa,’ an adaptation of the Diabetes Prevention Program, on weight loss compared to usual care.	South Africa	RCT	Adults with overweight or obese weight status (*N* = 494, majority women, mean age 68 years)	Type 2 Diabetes prevention	17-session group program facilitated by trained community health workers including videos, interactive activities, peer support and covered topics on goal setting, physical activity, diet and stress management. Facilitator training included 3-day interactive workshops and 8 weekly half-day experiential training, and supervision of intervention sessions.
Diriba et al. [36]; Diriba et al. [34]; Diriba et al. [35];	To examine the effects after two months of receiving a culturally tailored, family-supported, community-based diabetes self-management programme on:A. lycosylated hemoglobulin (HbA1c), blood pressure, body mass index and lipid profiles. B. perceived support status of people with diabetes and the family’s caregiver’s support behaviour. C. diabetes self-management behaviour and quality of life.	Ethiopia	Pilot RCT	Adults living with type 2 diabetes and family caregivers (*N* = 76 patient-caregiver dyads)	Type 2 diabetes	Six nurse-led educational and diabetes management sessions conducted face-to-face in a group setting for patient-caregiver dyads. Nurses received one-day training in the intervention and delivery. Educational workbook provided, demonstrations of blood glucose monitoring and videos on insulin self-injection and foot care.
Doumbia et al. [52];	To use a participatory approach to adapt the Diabetes Prevention Program [54] for delivery by community health workers in the Malian context, and to obtain feedback on the adapted sessions and assess their potential for promoting behavioural change with potential participants	Mali	Intervention adaptation description involving a focus group discussion and pilot testing of the intervention content with group feedback.	Adults living with or at risk of developing type 2 diabetes and community health workers. (N for focus groups = 19 Community health workers; N for group feedback = 45 people with or at risk of diabetes)	Type 2 diabetes	12 face-to-face, facilitator-led group sessions on physical activity, nutrition, and changing behaviour. Facilitators were community health workers who received training on the intervention. Sessions were conducted three times a week for four weeks.
Draper et al. [41];	To report on the process evaluation of the Healthy Conversation Skills approach, to identify implementation challenges and make recommendations for adaptations.	South Africa	Qualitative study with interviews, focus groups and facilitator debrief sessions	18–28-year-old women (*N* = 35)	Young women’s pre-conception health	18 facilitator-led individual sessions delivered on a monthly basis to promote behaviour change using the Healthy Conversation Skills approach. Facilitators were peers who were trained (28 training days for all intervention components) and had similar qualifications as community health workers.
Ernstzen et al. [42]	To explore the feasibility and acceptability of ePEEP (Patient Education Empowerment Programme) through the perspectives of individuals with chronic pain who participated in the programme	South Africa	Qualitative study with post-intervention interviews	Adults living with chronic pain (*N* = 6, all women)	Chronic pain	Telehealth intervention for six weeks with educational content and text messages sent and a weekly group videocall for collaborative discussion. The sessions were facilitator led; facilitators involved did not receive training as they had co-developed the intervention.
Fayehun et al. [49]	To examine the effect of a 10,000 steps per day target on glycaemic control of adults living with Type 2 diabetes after a 10-week period.	Nigeria	RCT	Adults living with type 2 diabetes aged 18–64 years, non-insulin dependent, with ability to walk. (*N* = 46)	Type 2 diabetes	10-week intervention to achieve a daily walking target goal using a pedometer. Telephone follow up calls at weeks 2, 6 and 10 and additional counselling support at weeks 4 and 8. Counselling and follow up calls were carried out by the study authors.
Grodensky et al. [53];	To describe the adapted motivational interviewing intervention to reduce the risk of onward HIV transmission among individuals with acute HIV infection and explore the feasibility of the intervention.	Malawi	Qualitative study using interviews with counsellors and participants	Adults living with HIV and intervention facilitators/counsellors (*N* = 2 counsellors and 14 intervention participants)	Secondary HIV prevention	Multi-component motivational interview-based intervention delivered by counsellors in four monthly sessions to reduce risky sexual behaviour. Counsellors had certificates in education, training and volunteering experience in HIV counselling and testing and received intensive training in motivational interviewing.
Gyamfi et al. [48]	To identify and describe the community health nurses’ perceptions of enablers and challenges faced with a task shifting strategy program for hypertension management.	Ghana	Qualitative study with interviews and focus group discussions	Nurses who participated in the intervention program (*N* = 27)	Hypertension	Nurse-led task shifting strategy for managing hypertension. Included counselling on medication adherence, physical activity, diet and weight loss. Delivered once every 3 months for a period of 12 months. Nurses received training in the WHO – Package of essential non-communicable disease intervention for primary care.
Ikolaba et al. [50];	To develop and test the feasibility of a community pharmacy person-centred goal-setting intervention for adults living with type 2 diabetes to improve clinical outcomes such as plasma glucose levels, waist circumference and body mass index.	Nigeria	Feasibility trial including questionnaires measuring self-reported outcomes (patient activation, medication adherence, and quality of life) and interviews with adults living with type 2 diabetes and pharmacists	Adults living with type 2 diabetes who self-managed their disease and pharmacists who delivered the intervention. (*N* = 70 participants completed intervention and outcome measures; N for interviews = 10 pharmacists and 15 adults living with type 2 diabetes)	Type 2 diabetes	Pharmacist-led intervention involving collaborative development of a diabetes care plan with the person living with diabetes. Six sessions that took place monthly over six months. The first and last session were delivered face-to-face and the remaining four sessions over the phone. Pharmacists attended a full-day online training workshop on the intervention and motivational interviewing.
Jackson et al. [43]	To determine the effectiveness of the Positive Living programme and a therapeutic relationship (relationship that forms between participant and research assistant resulting from repeated interaction during data collection), compared to the therapeutic relationship alone in managing pain (i.e. pain severity and pain interference).	South Africa	RCT	Rural amaXhosa women living with HIV (*N* = 49)	Pain management with HIV	6-week, peer-led group intervention comprising of education on living well with HIV, exercise and goal setting. Peer facilitators received 40 hours of training in the intervention and sessions took place weekly for two hours each.
*Muchiri [37]; Muchiri et al. [24]; Muchiri et al. [38];	A. To plan a tailored nutrition education programme for adults living with type 2 diabetes in a resource limited setting. B. To evaluate the effect of a nutrition education programme on glycated Hb (HbA1c), blood lipids, blood pressure, BMI and dietary behaviours in people living with type 2 diabetes at 6- and 12-months post-intervention. C. To investigate how a randomised controlled trial of an adapted diabetes nutrition education programme was received by participants in a tertiary setting.	South Africa	A. Qualitative study on needs assessment B. RCT C.Qualitative study – process evaluation with interviews and focus groups	A. 31 adults living with diabetes and 10 nursing professionalsB. Adults living with type 2 diabetes (*N* = 82)C. Adults with sub-optimally controlled (HbA1c of ≥8%) type 2 diabetes (*N* = 45)	Type 2 diabetes	three main intervention components − 8 weekly sessions on nutrition education, follow up sessions (four monthly meetings and two bi-monthly meetings), and vegetable gardening demonstrations incorporated into the group sessions. The sessions were led by a dietician and conducted in a group setting.
Ogunlana et al. [51];	To investigate the effect of a progressive goal attainment programme alongside conventional treatment on pain intensity, pain catastrophising, kinesiophobia, disability and self-efficacy at the end of 10 weeks.	Nigeria	Quasi-experimental study with experimental and control group sequentially assigned.	Adults living with non-specific lower back pain (*N* = 70)	Back pain	Ten weekly in-person sessions with a rehabilitation healthcare professional trained in delivering the intervention. Activity-based CBT intervention consisting of goal attainment techniques, activity and mobilisation strategies.
Parker et al. [44];	To explore patient satisfaction with, acceptability of, and the perceived success of a chronic pain management programme.	South Africa	Qualitative study with semi-structured interviews	Adults living with chronic pain (*N* = 14)	Chronic pain	Six session pain management programme led by physiotherapists in a group setting consisting of exercises, education, discussion, and relaxation. The sessions were conducted weekly, face-to-face in a hospital setting.
Saw et al. [45];	To explore the effects of physiotherapist-led exercise and education intervention on pain at 6 weeks, 12 weeks and 6 months.	South Africa	RCT	Adults living with osteoarthritis and on a waiting list to receive hip/knee arthroplasty (*N* = 74)	Osteoarthritis	Six sessions in a group setting, led by physiotherapists and included an exercise, education and relaxation component. The sessions were held weekly, for two hours each, at a hospital outpatient setting.
Vertue [46];	To determine the impact of a lifestyle intervention on the perception of diabetes control and perceived ability to adhere to lifestyle changes related to diet and exercise measured through a questionnaire and on HbA1c count at three months post-intervention.	South Africa	Pilot RCT	Adults living with Type 1 diabetes diagnosed in the last 12 months (*N* = 38)	Type 1 diabetes	Six sessions in a group setting, led by a facilitator who was a trained social worker. The programme was a multi-component lifestyle intervention with educational and practical activities.

*The amount of information on participant characteristics and intervention descriptions varied across the included studies.

### Quality assessment

Studies included in the review were mainly qualitative studies or randomised controlled trials. Overall, the methodological quality of each publication was good with 10 studies meeting all 5 quality criteria. The remaining publications were of moderate methodological quality and met at least three quality criteria. The main limitations that affected the quality of evidence were the incomplete reporting and lack of transparency of methods used and how findings were interpreted. From the qualitative studies, scores were weaker for the criteria ‘are the findings adequately derived from the data’ (criteria 1.3) with inadequate reporting in some publications. The randomised controlled trials tended to score less on the quality criteria ‘are outcome assessors blinded to the intervention provided’ (criteria 2.4). The quality assessment for individual publications can be found in supplementary file 2. All studies were retained for analysis.

### Use of goal setting components

Publications varied in the level of detail provided in relation to the goal setting component of the intervention (Table 3). Goal setting was most often a component of larger multi-component behaviour change interventions and was introduced or integrated into sessions focused on education or practical tasks related to self-management. For three interventions, goal setting was the main focus; one used the SMARTER tool for facilitating participant goal setting using the Healthy Conversations Skills approach [41], one used a pedometer and set a standardised goal for daily step counts for participants [49], and one used a progressive goal attainment programme in the context of rehabilitation for lower back pain [51]. Three interventions used the cognitive behavioural model [32,33,45,51], three used social cognitive theory [24,34–38,40], and four interventions used the SMART/SMARTER framework [32,33,41,46,50] to shape their use of goal setting. Eight interventions did not mention a theory or framework to shape or guide goal setting activities [31,39,42,43,47–49,52,53]. Interventions delivered in-person and those with a larger number of sessions tended to offer more support with personalising and reviewing goals and action plans. Personalisation of goals was achieved in different ways for individual v/s group interventions. With one-to-one interventions, facilitators provided support with identifying and personalising goals, developing action plans, identifying barriers and ways to overcome them, reviewing goals and motivating continued goal setting [31,39,47–51,53]. With interventions in group settings, facilitators provided information about goal setting and its importance, instruction about how to set goals and create action plans, and reflection and feedback on goal achievement [32,33,42,43]. Some discussion was reported amongst participants about goal setting challenges, successes, and learning from each other’s experience which was beneficial for formulating action and coping plans [32–36,40,46]. Personalisation took place through using examples from some participants to explain goal setting concepts or reviewing goals and asking other participants to reflect on and apply the learnings to their individual goals. Four interventions used written materials for the session, mainly worksheets or workbooks [40–42,51], and three interventions used logbooks or diaries to set and monitor goal practice at home [45,47,52].

**Table 3.** Goal setting components of interventions included in the review (*N* = 24 publications, 18 unique interventions).

Author	Theory or framework for goal setting	Goal setting activities during the intervention session	Goal setting stages targeted*	Goal setting adaptation to cultural context	Activities or support with goal setting after or in-between sessions (e.g. handouts, homework tasks, reminders)	Data on experience or engagement with goal setting component
Absetz et al. [31]; Van Olmen et al. [39];	Not mentioned	Peer group leaders encouraged participants to set lifestyle goals. This involved identifying existing healthy behaviours carried out by the participant and providing guidance on setting concrete and realistic goals and plans.	Preparation, Formulation of a goal, Follow up	Not mentioned	–	No
Appiah et al. [32]; Appiah et al. [33];	Cognitive behavioural model and SMART framework	Group discussion on the importance of setting goals. Group brainstorming activity on what goals are meaningful, how to achieve them, and what challenges they may face and how to overcome them. Facilitators explain SMART goals. Each participant is tasked with setting a SMART goal during the session and discussing what aspects of goal setting they find most challenging.	Preparation, Formulation of goal, Formulation of action plan, Coping planning	Not mentioned	Participants were asked to identify two things they value and make a plan to do them, reflect on the outcome, congratulate themselves for doing them, and think of how these make their life more meaningful.	Yes
Asante et al. [47];	–	Nurse facilitators provided information on diabetes self-management during the calls and assisted participants to set and evaluate individualised goals.	Preparation, Formulation of action plan, Follow up	Not mentioned	Diary to record call date and duration, personalised goals, action plans, and self-management challenges.	No
Catley et al. [40];	Social cognitive and problem-solving theory	Goal setting worksheets to support individualised goal setting linked to the program goals of weight loss and increase in physical activity completed during the session. Discussion of goal challenges and success during sessions.	Preparation, Formulation of goal, Formulation of action plan, Coping planning, Follow up	Videos and worksheets translated into isiXhosa, pauses built into the videos when there was an activity with participants (e.g. for personalising concepts and goal setting activities)	SMS messages to motivate behaviour change between sessions.	No
Diriba et al. [36]; Diriba et al. [34]; Diriba et al. [35];	Social cognitive theory	Goals were set and reviewed each week during the session. Sharing of experiences and group discussion between participants so that there was learning and motivation form peer’s experiences. Verbal appraisal and feedback was given by the facilitators. Family members were encouraged to co-attend and set goals together.	Preparation, Formulation of goal, Follow up	Culturally specific nutritional knowledge (e.g. traditional food recommendations and pictures). Intervention added family support element with food planning, preparation and self-care activities.	Handbook and fliers with content from intervention sessions.	No
Doumbia et al. [52];	Not specified (if same as DPP, then Social Cognitive Theory and problem solving)	Goal setting was integrated into the intervention content around creating a game plan for nutrition and exercise. Flipcharts and videos were used to educate participants on diet and physical activity. Role plays were used to illustrate spouses supporting each other and build confidence to introduce the program to other family members.	Preparation, Formulation of a goal, Formulation of an action plan, Coping planning	Adaptation of the game plan daily food log to make it suitable for low literacy levels with pictures of most common foods. Cultural aspects of diet considered e.g. added information about street food and at family celebrations and removed information about reading food menus.	Participants were encouraged to elicit family support with achieving goals through their game plan.	Very little
Draper et al.) [41];	Healthy Conversation Skills approach and SMARTER planning tool	Open discovery questions and the SMARTER tool to guide and structure goal setting. Person-centred goals, framed with facilitator support.	Preparation, Formulation of a goal, Formulation of an action plan, Coping Planning, Follow up	Not mentioned	–	Yes
Ernstzen et al. [42];	Not mentioned	Goal setting explanation and role-modelling video shown. Goal setting and reflection on goals integrated with exercise, nutrition, stress and medication intervention modules.	Preparation, Formulation of a goal, Follow up	Not mentioned	Facilitators and participants added to an online group to continue with discussion and support.	Yes
Fayehun et al. [49];	Not mentioned	Pre-set goal given to participants of 10,000 steps to achieve per day. Recorded on pedometer.	Formulation of goal, Coping planning, Follow up	No	Counselling and follow up by telephone to increase step count by 20% from baseline each week.	Yes
Grodensky et al. [53];	Not mentioned	Counsellors elicit strategies from participants to move towards safe behaviour and then negotiate an individualised behaviour change goal with participants for the next session. Participants initially set goals in relation to abstinence or safer sex, and HIV status disclosure to partners. In the later sessions more flexibility around goal setting topic was given to create an individualised plan and personalised goals.	Preparation, Formulation of a goal, Follow up	The intervention was condensed because of the high infectiousness of the acute HIV infection period. This included a shorter amount of information presented and reduced flexibility for participants in choosing topics and behaviour change goals in the initial sessions.	–	Yes
Gyamfi et al. [48];	Not mentioned	Community health nurses counselled participants in achieving lifestyle goals in relation to medication adherence, healthy diet, and physical activity using motivational interviewing techniques.	Preparation, Formulation of a goal, Coping planning	Not mentioned	–	Very little information
Ikolaba et al. [50];	SMART goals	Pharmacist-supported person-centred goal setting using motivational interviewing. Pharmacists started by asking what the participant perceived as the ‘problem’, then supported them in setting personal goals. These were reviewed during consultations and self-management support was provided using motivational interviewing.	Preparation, Formulation of a goal, Formulation of an action plan, Coping planning, Follow up	Not mentioned	–	Yes
Jackson et al. [43]	Not mentioned	In each session, there was an educational topic and group discussion, an action plan was developed, and action plans were reviewed in the next session. Peer support and facilitator support was used to develop action plans.	Preparation, Formulation of a goal, Formulation of an action plan, Coping planning, Follow up	Not mentioned	Workbooks were used in-between sessions and contained the exercise routine and other content covered in the intervention sessions.	No
Muchiri (2013) [37]; Muchiri et al. [24]; Muchiri et al. [38];	Social cognitive theory	Goal setting was integrated in each session. Participants chose dietary behaviour goals in relation to specific topics to practice during the week. Peer support and positive reinforcement was given for goal achievement.	Preparation, Formulation of a goal, Formulation of an action plan, Coping Planning, Follow up	Not mentioned	Printed education materials in form of handouts (pamphlet and fridge/wall flyer) on diabetes basics and healthy eating.	Yes
Ogunlana et al. [51]	Cognitive behaviour therapy	Activity goals are framed with the help of the facilitator and reviewed in subsequent sessions. Techniques to deal with fears of engaging in activity and rehabilitation are taught.	Preparation, Formulation of a goal, Formulation of an action plan, Coping Planning, Follow up	Not mentioned	Workbook to take home and review activities at each session.	No
Parker et al. [44]	Cognitive Behaviour Therapy	Goals were selected at the end of the discussion and problem-solving section and related to the topic discussed. Weekly feedback on goal achievement was given during the sessions.	Preparation, Formulation of a goal, Follow up	Not mentioned	–	Very little information
Saw et al. [45]	Social learning and cognitive behavioural approaches	Exercise and activity goals were set on a weekly basis during the sessions and recorded in a workbook.	Preparation, Formulation of a goal, Formulation of an action plan	Yes – translation into local languages	Workbook to facilitate between session practice and monitoring of goal achievement.	No
Vertue (2016) [46]	SMART goals framework	Education about goal setting, group activity to create SMART goals, then participants set their own goal and reviewed it the next week.	Preparation, Formulation of a goal, Follow up	Not mentioned	–	Very little information

*Goal setting stages are described and applied according to Lenzen et al’s (2017) review on disentangling goal setting and action planning [55].

Two interventions used standardised goal targets [49,53], and the rest supported participants to set personal goals. Some interventions encouraged participants to set goals relevant to the topics covered in the intervention [24,37,38,40,45,48,51–53]. While most interventions included tasks that targeted each of the five stages of goal setting; the least used stages were developing action and coping plans. Interventions that had a longer duration offered more opportunity for addressing all stages, especially for goal attainment evaluation and follow up. In 17 out of the 18 interventions, the goal setting activity was preceded by reflection on current behaviour and identification of areas for goal setting. For 14 interventions (78%), goals were revisited and reviewed in subsequent sessions [24,31,34–40,42–44,46,47,49–51,53]. In six interventions, facilitators encouraged participants to think about the steps they could take to achieve their goals and formulate explicit action plans [24,32,33,37,38,43,50–52] and in two interventions, participants developed action plans at home [45,47]. In eight interventions, participants developed coping plans during the intervention session through discussion with the facilitator on motivators and barriers to following the action plan and how to address these barriers [32,33,40,43,44,49–51]. Very few studies reported adapting goal setting to the cultural context. Five interventions adapted the goal setting tasks to the local cultural or economic context of participants by translating intervention materials to local languages, using examples of local foods, simplifying language, using pictures, and by involving family in the intervention sessions and encouraging family members to offer personal support in the planning and execution of goals at home [34–36,40,52,53].

### Experience and engagement with goal setting activities

Few publications (7/24) related to seven unique interventions contained details around experience of and engagement with goal setting from a participant perspective [32,41,42,44,46,50,53] and few publications (6/24) related to five unique interventions included facilitator feedback on the use of goal setting [37,39,41,50,53]. Findings were grouped into the three themes outlined below.

#### Value of goal setting

Participants reported that their experience of using goal setting was positive and empowering [32,41,46,50,53]. Goal setting helped to put self-management tasks into practice and integrate them into their daily lives. Facilitators observed that the goal setting helped make self-management advice more actionable and self-monitoring encouraged people to stick to their goals [50,53]. Having goals and action plans were experienced as both comforting and rewarding for participants [50] and useful in helping them commit to action [53].

#### Factors that facilitate goal setting

Personalisation of goals was important for goal setting, as it allowed participants’ sense of control, motivation and commitment to goal achievement to increase [37,42,50,53]. In one group intervention, personalised health behaviour goals were created by linking to people’s values and broader life goals [32]. Facilitator support with personalising goals was found to be helpful in one-to-one interventions [42,50,53] and facilitators found understanding participants’ level of confidence with goal achievement useful for tailoring support [53]. Group discussions and sharing experiences with peers enabled participants to gain more confidence to pursue their goals [32,39]. One study highlighted how integrating demonstrations or examples of setting SMART goals helped participants with acquiring goal setting skills [32]. Facilitators and participants in another intervention reported that practicing short-term goals within the intervention timeframe enabled participants to set longer term goals and view them as more manageable [50].

#### Challenges with goal setting

In some interventions, facilitators identified challenges with goal setting activities, which in some cases led to facilitators suggesting goals or conducting goal setting as a group activity [37,39]. One publication recommended following up the group sessions with individual counselling to boost goal setting and maintain behaviour change [37]. Low literacy and language barriers were also identified as challenges when setting SMARTER goals and selecting goals from a pre-specified behaviour change list [37,41]. The SMARTER tool in particular was found to be too structured and complicated for both facilitators and participants [41]. Interventions generally took between 4 and 10 sessions which was sometimes too short for reviewing goals that participants put into practice [42].

## Discussion

Our review aimed to synthesise existing literature on the different ways goal setting was used and has provided a summary of how goal setting has been used in 18 interventions for chronic disease prevention and management in SSA. The 18 interventions directed at diabetes prevention and management, pain management, hypertension management, prevention of mental health problems, women’s pre-conception health, and HIV risk reduction and treatment have used a variety of goal-setting frameworks and strategies. Interventions were often facilitator-led and conducted in group settings. Goal setting tasks for most interventions targeted the preparation, goal formulation, and follow up stages and fewer interventions targeted the action plan and coping plan formulation stages. Participants and facilitators found goal setting useful for translating self-management advice into practice and for enhancing motivation for behaviour change. Challenges included delivering structured goal setting activities (e.g. SMARTER goals), with participant language and literacy levels acting as barriers, and a need for personalising goals to the participants’ life story and context.

Consistent and adequate reporting of goal setting strategies used in interventions is necessary to make evidence-based recommendations for delivering goal setting interventions in SSA, but in our review, studies displayed variable amounts of information on the use of goal setting as intervention components. Some studies in this review did not provide sufficient detail on the action planning and coping planning stages involved in goal setting. Information on the use of frameworks or theories was also limited, as well as reporting on the engagement and experience with goal setting. Publications typically reported engagement or effectiveness of the intervention overall. Using checklists such as the template for intervention description and replication (TIDieR) and checklist for group-based behaviour interventions can help improve transparency of information around intervention components and content [56,57]. As goal setting is a common component of behaviour change interventions, detail about this component and standardised reporting is necessary to enable drawing conclusions about this as a behaviour change technique and learning from best practice. In addition to using checklists like TiDieR, we propose a list of additional items to consider when reporting goal setting intervention components (Table 4).

**Table 4.** Proposed checklist for reporting goal setting intervention components.

*Goal setting theory or framework*	✓ Is the goal setting component of the intervention influenced by a particular behaviour change theory? ✓ Was a particular goal setting framework used (e.g. SMART goals)?
*Goal setting activity*	✓ How were the goal setting activities carried out during the intervention sessions (e.g. written activity, group discussion, self-reflection, filling in a questionnaire, facilitator support provided)
*Goal setting stages*	✓ Preparation – Did participants engage in any related activities prior to setting goals? ✓ Formulating a goal – Did participants make explicit goals? ✓ Formulating an action plan – Were action plans made to achieve these goals and were these made explicit? ✓ Coping planning – Were there any activities to identify potential barriers to the action plan and ways to overcome them? ✓ Follow up – Did participants work on achieving their goals or were they supported to achieve their goals and carry out their action plans?
*Adapting materials to cultural context*	✓ Was the structure of the goal setting activities adapted or modified to participants’ cultural context? ✓ Was the language or activity content adapted to the participants’ cultural context?
*Support between intervention sessions, homework, materials provided*	✓ Did participants receive any materials to support them with goal setting and goal achievement in-between/beyond intervention sessions (e.g. handouts or workbooks, logbooks or diaries)? ✓ Were participants given any reminders or support in-between intervention sessions (e.g. SMS prompts or follow up calls)?
*Goal setting evaluation*	✓ Was engagement with the goal setting component measured? ✓ Was success at achieving goals measured? ✓ Was participant or intervention facilitator experience of using goal setting captured?

Our review findings also point to the importance of understanding the lived experience and context of people living in SSA and of encouraging personalisation of goal framing to facilitate participant engagement. Goal setting in an African context needs to blend universal principles with deep cultural understanding to navigate complex realities. Findings from two studies indicated that the socio-economic context and life-history need to be considered to allow participants to see health-related goals as relevant and manageable and to increase confidence in their ability to achieve these goals [33,41]. This aligns with other research where participants in chronic disease self-management group programmes reported the importance of aligning goal setting objectives with their own values to increase their motivation and engagement [58]. Cultural adaptation is also important for personalising goals. In SSA, factors within different communities such as socio-economic status, power dynamics and gender or family roles may affect individual goal setting. This needs to be considered when designing goal setting tasks or personalising goals and action plans which will require a realistic assessment of what is feasible. In our review, studies also indicated that personalising goals was experienced as useful [37,42,50,53]. It is, therefore, important to integrate either independent or facilitator-supported personalisation of goals to enhance engagement with goal setting in interventions. Facilitators should be trained to be familiar with participants’ cultural context or be from the same communities as the participants to be able to support them with goal setting.

Almost all interventions in our review included a preparation stage where goal setting was introduced, linked with the self-management topic being discussed, and participants were encouraged to reflect on current behaviour and areas for change. Interventions also included a follow up stage where goals were reviewed and feedback and reflections from facilitators or group participants helped refine and set new goals [24,31,34–40,42–44,46,47,49–51,53]. Including and allocating enough time for these stages in interventions can help ease participants into goal setting activities and give them the support and guidance they need to work towards these goals in their daily lives.

**Figure 2.**
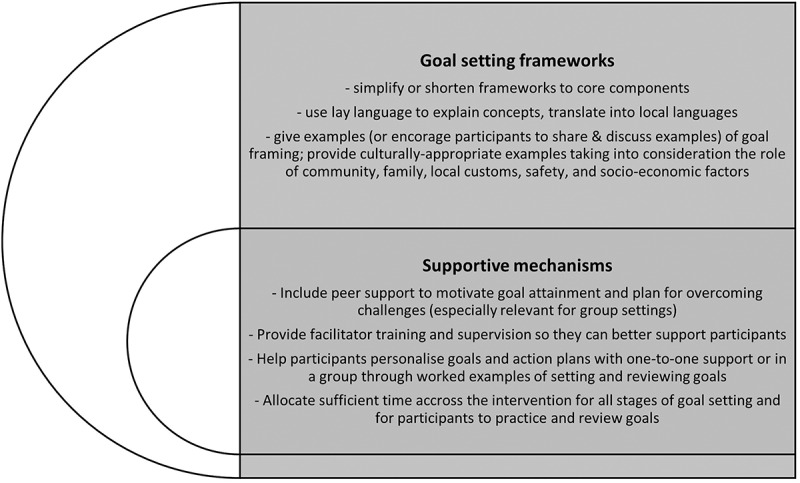
Recommendations for using goal setting for behaviour change interventions in SSA.

Our findings also point to the challenges with low literacy and language barriers that can hinder participation in goal setting activities. Some suggestions to overcome these barriers include simplifying frameworks such as SMARTER goals or slowly introducing the different criteria for goal framing, using fewer technical terms, lay language and prompts that can be easily translated into local languages [41]. However, none of the studies in the review provided evidence of success of a simpler version of the SMARTER framework. Providing examples or demonstrations of how to frame goals according to a particular framework could also be advised. In our review, participants who received the intervention in a group setting found sharing and learning from others’ experiences helpful, particularly with respect to overcoming challenges and improving confidence [32,39]. Facilitators often received training to deliver the intervention, but challenges were identified with respect to the use of frameworks and goal setting with individual participants [37,39,41]. It may be useful for facilitators to receive ongoing support specifically with goal setting intervention components and to provide refresher training and ongoing supervision for facilitators during intervention delivery to cope with challenges as they arise [59,60]. Our recommendations based on the findings of this review include simplifying goal setting frameworks, using colloquial local language and translations when framing goals, and utilising peer support to motivate and practice goal setting (see Figure 2). These recommendations could also be used by public health initiatives and established programmes in the region such as WHO package of essential noncommunicable (PEN) disease interventions for primary care and HEARTS [61,62] or smaller scale community-based initiatives to effectively integrate goal setting into existing educational and behavioural interventions for NCDs.

In our ongoing couples-focused intervention in South Africa (Diabetes Together), the SMARTER tool for goal setting was simplified based on feedback from participants and facilitators after the first workshop. The simplified version was still not effective in the second workshop but worked well within the individual counselling sessions. It needs to be further adapted to simplify the language, integrate more examples, and use less technical terms for delivery in a group setting. It may be helpful to include more time within the intervention delivery schedule to introduce goal setting and its relevance to diabetes management, emphasise goal personalisation and identify underlying reasons or motivations for participant goals. It may also be worth including follow up sessions where goals are reviewed and creating a space where participants can learn from each other’s experiences. The studies included in this review did not include goal setting with partners or couples, except for one intervention where family members assisted participants with goal execution [34–36]. It is still unclear, however, how each partner in the couple can be involved in the goal setting process and activities. Some literature suggests that couples can set joint goals and work towards achieving these [63,64]; whereas other literature indicates that the spouses’ role is to assist participants with achieving their goals [65,66].

### Strengths and limitations

Our review included studies with varying research methods and designs to offer a comprehensive picture of goal setting for chronic disease interventions in SSA. Although this heterogeneity was viewed as a strength, it did not allow us to draw definite conclusions about effective goal setting strategies and delivery. Inadequate reporting of the goal setting components also limited the data that could be synthesised. Future research could examine the influence of goal setting on intervention engagement, behaviour change, and other health outcomes.

Our search strategy may not have identified all relevant studies using goal setting intervention components, as ‘goal setting’ may be a term used in the research article but not in the title or abstract unless it is a main component of the intervention. This risk was mitigated by using subject headings and other search terms in addition to ‘goal setting’ around self-management and self-care.

## Conclusion

In this review of the use of goal setting within chronic disease prevention and management in SSA, interventions were often facilitator-led and conducted in group settings. Goal setting intervention components were informed by a variety of frameworks and involved a range of tasks. Participants reported goal setting as useful for translating self-management into practice but also encountered challenges related to language and literacy levels. The included studies ranged from high to moderate in terms of methodological quality but did not always report adequate details of the goal setting intervention components. Future studies should routinely incorporate and report information around goal setting theory or frameworks, the stages of goal setting around action planning and coping planning and how goal setting activities were adapted to the specific culture and population.

## Supplementary Material

Supplementary file.docx

PRISMA checklist.docx

## Data Availability

The data that support the findings of this review are openly available in the included articles of this review.
