# Supplementary material for: How is goal setting used in interventions for chronic disease prevention and management in sub-Saharan Africa? A systematic review and narrative synthesis
Source: Glob Health Action. 2026 Jan 12;19(1):2608423. doi: 10.1080/16549716.2025.2608423 (PMC12798663; doi:10.1080/16549716.2025.2608423)
Supplement: Supplementary file.docx [file ZGHA_A_2608423_SM7674.docx]

# Supplementary file 1

**Caption: Search strategy and terms in the different databases**

Medline Ovid

| Number | Search Term(s) |
| --- | --- |
| 1 | Goal$.mp |
| 2 | Goal-setting$.mp |
| 3 | Action plan$.mp |
| 4 | Coping plan$.mp |
| 5 | Review goal$.mp |
| 6 | Self management/ |
| 7 | Self care/ |
| 8 | 1 OR 2 OR 3 OR 4 OR 5 OR 6 OR 7 |
| 9 | Africa/ |
| 10 | Sub-saharan Africa$.mp |
| 11 | (Cameroon or "Central African Republic" or Chad or "Democratic Republic of the Congo" or "Equatorial Guinea" or Gabon or "Sao Tome and Principe" or Burundi or Djibouti or Eritrea or Ethiopia or Kenya or Rwanda or Somalia or "South Sudan" or Sudan or Tanzania or Uganda or Angola or Botswana or Lesotho or Malawi or Mozambique or Namibia or "South Africa" or Swaziland or Eswatini or Zambia or Zimbabwe or Benin or "Burkina Faso" or "Cabo Verde" or Cote d'Ivoire or "Ivory Coast" or Gambia or Ghana or Guinea or Guinea-Bissau or Liberia or Mali or Mauritania or Niger or Nigeria or Senegal or "Sierra Leone" or Togo or Madagascar).mp |
| 12 | 9 OR 10 OR 11 |
| 13 | Chronic disease$.mp |
| 14 | Chronic illness$.mp |
| 15 | Chronic condition$.mp |
| 16 | Long-term condition$.mp |
| 17 | Chronic heart disease$.mp |
| 18 | Diabetes.mp |
| 19 | Hypertension.mp |
| 20 | Chronic depression.mp |
| 21 | Chronic lung disease$.mp |
| 22 | Chronic pain.mp |
| 23 | Asthma/ |
| 24 | Pulmonary disease, chronic obstructive/ |
| 25 | Diabetes/ |
| 26 | Hypertension/ |
| 27 | Chronic Pain/ |
| 28 | Smoking cessation.mp |
| 29 | Alcohol$.mp |
| 30 | Weight loss.mp |
| 31 | Physical activit$.mp |
| 32 | Exercise$.mp |
| 33 | Diet$.mp |
| 34 | Lifestyle$.mp |
| 35 | Behav$ chang$.mp |
| 36 | Health Promotion/ |
| 37 | 13 OR 14 OR 15 OR 16 OR 17 OR 18 OR 19 OR 20 OR 21 OR 22 OR 23 OR 24 OR 25 OR 26 OR 27 OR 28 OR 29 OR 30 OR 31 OR 32 OR 33 OR 34 OR 35 OR 36 |
| 38 | Intervention$ |
| 39 | Program$ |
| 40 | Service$ |
| 41 | Care$ |
| 42 | 38 OR 39 OR 40 OR 41 |
| 43 | African American$.mp |
| 44 | Infant$.mp |
| 45 | Child$.mp |
| 46 | Adolescen$.mp |
| 47 | Gestation$.mp |
| 48 | Pregnan$.mp |
| 49 | 38 OR 39 OR 40 OR 41 OR 42 OR 43 |
| 50 | 8 AND 12 AND 37 AND 42 |
| 51 | 50 NOT 49 |
| 52 | Limit 46 to English language |

PsycINFO

| Number | Search term |
| --- | --- |
| S1 | Goal* |
| S2 | “goal setting” |
| S3 | “action plan*” |
| S4 | “coping plan*” |
| S5 | “review goal*” |
| S6 | “self manag*” |
| S7 | “self care” |
| S8 | DE “Goal Setting” |
| S9 | DE “Self-management” |
| S10 | DE “self-care” |
| S11 | S1 OR S2 OR S3 OR S4 OR S5 OR S6 OR S7 OR S8 OR S9 OR S10 |
| S12 | Africa* |
| S13 | "Sub-Saharan Africa*" OR "Cameroon" OR "Central African Republic" OR "Chad" OR "Congo" OR "Democratic Republic of the Congo" OR "Equatorial Guinea" OR "Gabon" OR "Sao Tome and Principe" OR "Sao Tome" OR "Burundi" OR "Djibouti" OR "Eritrea" OR "Ethiopia" OR "Kenya" OR "Rwanda" OR "Somalia" OR "South Sudan" OR "Sudan" OR "Tanzania" OR "Uganda" OR "Angola" OR "Botswana" OR "Lesotho" OR "Malawi" OR "Mozambique" OR "Namibia" OR "South Africa" OR "Swaziland" OR "Eswatini" OR "Zambia" OR "Zimbabwe" OR "Benin" OR "Burkina Faso" OR "Cabo Verde" OR "Cote d’Ivoire" OR "Ivory Coast" OR "Gambia" OR "Ghana" OR "Guinea" OR "Guinea-Bissau" OR "Liberia" OR "Mali" OR "Mauritania" OR "Niger" OR "Nigeria" OR "Senegal" OR "Sierra Leone" OR "Togo" OR "Madagascar" |
| S14 | S12 OR S13 |
| S15 | “chronic disease*” |
| S16 | “Chronic illness*” |
| S17 | “chronic condition*” |
| S18 | “long term illness*” |
| S19 | “chronic heart disease*” |
| S20 | “chronic lung disease*” |
| S21 | Diabetes |
| S22 | Hypertension |
| S23 | “chronic pain” |
| S24 | “chronic depression” |
| S25 | DE “asthma” |
| S26 | DE “hypertension” |
| S27 | DE “Diabetes Mellitus” |
| S28 | DE “Chronic Obstructive Pulmonary Disease” |
| S29 | DE “Chronic pain” |
| S30 | “smoking cessation” |
| S31 | “alcohol*” |
| S32 | “weight loss” |
| S33 | “physical activit*” |
| S34 | “exercise*” |
| S35 | “diet*” |
| S36 | “lifestyle*” |
| S37 | “behav* chang*” |
| S38 | DE “Health Promotion” |
| S39 | DE “Preventative health behavior” |
| S40 | S15 OR S16 OR S17 OR S18 OR S19 OR S20 OR S21 OR S22 OR S23 OR S24 OR S25 OR S26 OR S27 OR S28 OR S29 OR S30 OR S31 OR S32 OR S33 OR S34 OR S35 OR S36 OR S37 OR 38 OR 39 |
| S41 | “intervention*” |
| S42 | “program*” |
| S43 | “service” |
| S44 | “care” |
| S45 | S41 OR S42 OR S43 OR S44 |
| S46 | “African American*” |
| S47 | “infant*” |
| S48 | “child*” |
| S49 | “adolescen*” |
| S50 | “gestation*” |
| S51 | “pregnan*” |
| S52 | S41 OR S42 OR S43 OR S44 OR S45 OR S46 |
| S53 | S11 AND S14 AND S40 AND S45 |
| S54 | S53 NOT S52 |
| S55 | Filter results in english |

CINAHL Plus

| Number | Search term |
| --- | --- |
| S1 | Goal* |
| S2 | “goal setting” |
| S3 | “action plan*” |
| S4 | “coping plan*” |
| S5 | “review goal*” |
| S6 | “patient education” |
| S7 | “self manag*” |
| S8 | “self care” |
| S9 | MH “Patient Education” |
| S10 | MH “Goal Setting” |
| S11 | MH “Self-management” |
| S12 | MH “self-care” |
| S13 | S1 OR S2 OR S3 OR S4 OR S5 OR S6 OR S7 OR S8 OR S9 OR S10 OR S11 OR S12 |
| S14 | MH “Africa” |
| S15 | "Sub-Saharan Africa*" OR "Cameroon" OR "Central African Republic" OR "Chad" OR "Congo" OR "Democratic Republic of the Congo" OR "Equatorial Guinea" OR "Gabon" OR "Sao Tome and Principe" OR "Sao Tome" OR "Burundi" OR "Djibouti" OR "Eritrea" OR "Ethiopia" OR "Kenya" OR "Rwanda" OR "Somalia" OR "South Sudan" OR "Sudan" OR "Tanzania" OR "Uganda" OR "Angola" OR "Botswana" OR "Lesotho" OR "Malawi" OR "Mozambique" OR "Namibia" OR "South Africa" OR "Swaziland" OR "Eswatini" OR "Zambia" OR "Zimbabwe" OR "Benin" OR "Burkina Faso" OR "Cabo Verde" OR "Cote d’Ivoire" OR "Ivory Coast" OR "Gambia" OR "Ghana" OR "Guinea" OR "Guinea-Bissau" OR "Liberia" OR "Mali" OR "Mauritania" OR "Niger" OR "Nigeria" OR "Senegal" OR "Sierra Leone" OR "Togo" OR "Madagascar" |
| S16 | S14 OR S15 |
| S17 | “chronic disease*” |
| S18 | “Chronic illness*” |
| S19 | “chronic condition*” |
| S20 | “long term illness*” |
| S21 | “chronic heart disease*” |
| S22 | “chronic lung disease*” |
| S23 | Diabetes |
| S24 | Hypertension |
| S25 | “chronic pain” |
| S26 | “chronic depression” |
| S27 | MH “asthma” |
| S28 | MH “hypertension” |
| S29 | MH “Diabetes Mellitus” |
| S30 | MH “Pulmonary Disease, Chronic Obstructive” |
| S31 | MH “Chronic pain” |
| S32 | “smoking cessation” |
| S33 | “alcohol*” |
| S34 | “weight loss” |
| S35 | “physical activit*” |
| S36 | “exercise*” |
| S37 | “diet*” |
| S38 | “lifestyle*” |
| S39 | “behav* chang*” |
| S40 | MH “Health Promotion” |
| S41 | S17 OR S18 OR S19 OR S20 OR S21 OR S22 OR S23 OR S24 OR S25 OR S26 OR S27 OR S28 OR S29 OR S30 OR S31 OR S32 OR S33 OR S34 OR S35 OR S36 OR S37 OR S38 OR S39 OR S40 |
| S42 | “intervention*” |
| S43 | “program*” |
| S44 | “service” |
| S45 | “care” |
| S46 | S42 OR S43 OR S44 OR S45 |
| S47 | “African American*” |
| S48 | “infant*” |
| S49 | “child*” |
| S50 | “adolescen*” |
| S51 | “gestation*” |
| S52 | “pregnan*” |
| S53 | S42 OR S43 OR S44 OR S45 OR S46 OR S47 |
| S54 | S13 AND S16 AND S41 AND S46 |
| S55 | S54 NOT S53 |
| S56 | Filter results in english |

Web of Science:

| Number | Search term(s) |
| --- | --- |
| #1 | TS=goal* |
| #2 | TS=”goal setting” |
| #3 | TS=”action plan*” |
| #4 | TS=”coping plan*” |
| #5 | TS=”review goal*” |
| #6 | TS=self manag*” |
| #7 | TS=”self care” |
| #8 | #1 OR #2 OR #3 OR #4 OR #5 OR #6 OR #7 |
| #9 | TS=”Africa” |
| #10 | TS=("Sub-Saharan Africa*" OR "Cameroon" OR "Central African Republic" OR "Chad" OR "Congo" OR "Democratic Republic of the Congo" OR "Equatorial Guinea" OR "Gabon" OR "Sao Tome and Principe" OR "Sao Tome" OR "Burundi" OR "Djibouti" OR "Eritrea" OR "Ethiopia" OR "Kenya" OR "Rwanda" OR "Somalia" OR "South Sudan" OR "Sudan" OR "Tanzania" OR "Uganda" OR "Angola" OR "Botswana" OR "Lesotho" OR "Malawi" OR "Mozambique" OR "Namibia" OR "South Africa" OR "Swaziland" OR "Eswatini" OR "Zambia" OR "Zimbabwe" OR "Benin" OR "Burkina Faso" OR "Cabo Verde" OR "Cote d’Ivoire" OR "Ivory Coast" OR "Gambia" OR "Ghana" OR "Guinea" OR "Guinea-Bissau" OR "Liberia" OR "Mali" OR "Mauritania" OR "Niger" OR "Nigeria" OR "Senegal" OR "Sierra Leone" OR "Togo" OR "Madagascar") |
| #11 | #10 OR #9 |
| #12 | TS=”chronic disease*” |
| #13 | TS=”chronic illness*” |
| #14 | TS=”chronic condition*” |
| #15 | TS=”long-term condition*” |
| #16 | TS=”chronic heart disease*” |
| #17 | TS=”chronic lung disease*” |
| #18 | TS=”diabetes” |
| #19 | TS=”hypertension” |
| #20 | TS=”chronic pain” |
| #21 | TS=”chronic depression” |
| #22 | TS=”asthma” |
| #23 | TS=”pulmonary disease, chronic obstructive” |
| #24 | TS=”smoking cessation” |
| #25 | TS=”alcohol*” |
| #26 | TS=”weight loss” |
| #27 | TS=”physical activit*” |
| #28 | TS=”exercise*” |
| #29 | TS=”diet*” |
| #30 | TS=”lifestyle*” |
| #31 | TS=”behav* change” |
| #32 | TS=”health promotion” |
| #33 | #12 OR #13 OR #14 OR #15 OR #16 OR #17 OR #18 OR #19 OR #20 OR #21 OR #22 OR #23 OR #24 OR #25 OR #26 OR #27 OR #28 OR #29 OR #30 OR #31 OR #32 |
| #34 | TS=”intervention” |
| #35 | TS=”program*” |
| #36 | TS=”care” |
| #37 | TS=”service” |
| #38 | #34 OR #35 OR #36 OR #37 |
| #39 | TS=”African American*” |
| #40 | TS=”infant*” |
| #41 | TS=”child*” |
| #42 | TS=”adolescen*” |
| #43 | TS=”gestation*” |
| #44 | TS=”pregnan*” |
| #45 | #39 OR #40 OR #41 OR #42 OR #43 OR #44 |
| #46 | #38 AND #33 AND #10 AND #8 |
| #47 | #46 NOT #45 |
| #48 | Filter #47 to English language only |

Global Index Medicus:

(tw:(Goal OR "goal setting" OR "self management" OR "self care" OR "action plan" OR "coping plan" OR "review goal" OR "patient education" OR "health education")) AND (tw:(Africa OR "sub-saharan Africa" OR "Cameroon" OR "Central African Republic" OR "Chad" OR "Congo" OR "Democratic Republic of the Congo" OR "Equatorial Guinea" OR "Gabon" OR "Sao Tome and Principe" OR "Sao Tome" OR "Burundi" OR "Djibouti" OR "Eritrea" OR "Ethiopia" OR "Kenya" OR "Rwanda" OR "Somalia" OR "South Sudan" OR "Sudan" OR "Tanzania" OR "Uganda" OR "Angola" OR "Botswana" OR "Lesotho" OR "Malawi" OR "Mozambique" OR "Namibia" OR "South Africa" OR "Swaziland" OR "Eswatini" OR "Zambia" OR "Zimbabwe" OR "Benin" OR "Burkina Faso" OR "Cabo Verde" OR "Cote d’Ivoire" OR "Ivory Coast" OR "Gambia" OR "Ghana" OR "Guinea" OR "Guinea-Bissau" OR "Liberia" OR "Mali" OR "Mauritania" OR "Niger" OR "Nigeria" OR "Senegal" OR "Sierra Leone" OR "Togo" OR "Madagascar")) AND (tw:("chronic disease" OR "chronic illness" OR "chronic condition" OR "long term condition" OR diabetes OR hypertension OR "chronic heart disease" OR "chronic depression" OR "chronic pain" OR asthma OR "chronic lung disease" OR "chronic obstructive pulmonary disease" OR "weight loss" OR alcohol OR "smoking cessation" OR "physical activity" OR exercise OR diet OR lifestyle OR "behaviour change" OR "health promotion"))

SCIELO:

(goal OR "goal setting" OR "self management" OR "self care" OR "action plan" OR "coping plan" OR "review goal" OR "self management" OR "self care" OR "health education" OR "patient education") AND (Africa OR "sub-saharan africa" OR "Cameroon" OR "Central African Republic" OR "Chad" OR "Congo" OR "Democratic Republic of the Congo" OR "Equatorial Guinea" OR "Gabon" OR "Sao Tome and Principe" OR "Sao Tome" OR "Burundi" OR "Djibouti" OR "Eritrea" OR "Ethiopia" OR "Kenya" OR "Rwanda" OR "Somalia" OR "South Sudan" OR "Sudan" OR "Tanzania" OR "Uganda" OR "Angola" OR "Botswana" OR "Lesotho" OR "Malawi" OR "Mozambique" OR "Namibia" OR "South Africa" OR "Swaziland" OR "Eswatini" OR "Zambia" OR "Zimbabwe" OR "Benin" OR "Burkina Faso" OR "Cabo Verde" OR "Cote d’Ivoire" OR "Ivory Coast" OR "Gambia" OR "Ghana" OR "Guinea" OR "Guinea-Bissau" OR "Liberia" OR "Mali" OR "Mauritania" OR "Niger" OR "Nigeria" OR "Senegal" OR "Sierra Leone" OR "Togo" OR "Madagascar") AND ("chronic disease" OR "chronic illness" OR "chronic condition" OR "long term condition" OR diabetes OR hypertension OR "chronic heart disease" OR "chronic depression" OR "chronic pain" OR Asthma OR "weight loss" OR "physical activity" OR exercise OR diet OR lifestyle OR "behaviour change" OR "health promotion")

Proquest for grey literature:

noft(“goal setting” OR “action plan” OR “coping plan” OR “self manag*” OR "self care") AND noft(Africa* OR "Sub-Saharan Africa*" OR "Cameroon" OR "Central African Republic" OR "Chad" OR "Congo" OR "Democratic Republic of the Congo" OR "Equatorial Guinea" OR "Gabon" OR "Sao Tome and Principe" OR "Sao Tome" OR "Burundi" OR "Djibouti" OR "Eritrea" OR "Ethiopia" OR "Kenya" OR "Rwanda" OR "Somalia" OR "South Sudan" OR "Sudan" OR "Tanzania" OR "Uganda" OR "Angola" OR "Botswana" OR "Lesotho" OR "Malawi" OR "Mozambique" OR "Namibia" OR "South Africa" OR "Swaziland" OR "Eswatini" OR "Zambia" OR "Zimbabwe" OR "Benin" OR "Burkina Faso" OR "Cabo Verde" OR "Cote d’Ivoire" OR "Ivory Coast" OR "Gambia" OR "Ghana" OR "Guinea" OR "Guinea-Bissau" OR "Liberia" OR "Mali" OR "Mauritania" OR "Niger" OR "Nigeria" OR "Senegal" OR "Sierra Leone" OR "Togo" OR "Madagascar") AND noft(“Chronic disease” OR “chronic illness” OR “chronic condition” OR “long term condition” OR “chronic heart disease” OR “diabetes” OR “chronic depression” OR “hypertension” OR “chronic lung disease” OR “chronic pain” OR "asthma" OR "COPD" OR "smoking cessation" OR "alcohol*" OR "weight loss" OR "physical activit*" OR "exercise*" OR "diet*" OR "lifestyle*" OR "behav* change" OR "health promotion") AND noft("intervention*" OR "program*" OR "care" OR "Service") NOT noft("african american*" OR "infant*" OR "child*" OR "adolescen*" OR "gestation*" OR "pregnan*")

# Supplementary file 2

***Table: Quality assessment of included publications (n=24)***

| Authors (year) | Mixed Methods Appraisal Tool criteria | | | | |
| --- | --- | --- | --- | --- | --- |
| Qualitative studies | 1.1 | 1.2 | 1.3 | 1.4 | 1.5 |
| Absetz et al, (2020) | Yes | Yes | Can’t tell | Yes | Yes |
| Appiah et al, (2020) | Yes | Yes | Can’t tell | Can’t tell | Yes |
| Appiah et al, (2021) | Yes | Yes | Yes | Yes | Yes |
| Draper et al, (2022) | Yes | Yes | Yes | Yes | Yes |
| Ernstzen et al, (2022) | Yes | Yes | Yes | Yes | No |
| Grodensky et al, (2018) | Yes | Yes | Yes | Yes | Yes |
| Gyamfi et al, (2020) | Yes | Yes | Yes | Yes | Yes |
| Muchiri et al, (2013) | Yes | Yes | Yes | Yes | Yes |
| Muchiri et al, (2023) | Yes | Yes | Yes | Yes | Yes |
| Parker et al, (2009) | Yes | No | Can’t tell | Yes | Yes |
| Van Olmen et al, (2022) | Yes | Yes | Can’t tell | No | Yes |
| Randomised controlled trials | 2.1 | 2.2 | 2.3 | 2.4 | 2.5 |
| Asante et al, (2020) | Yes | Yes | Yes | Can’t tell | Yes |
| Catley et al, (2022) | Yes | Yes | Yes | No | Yes |
| Diriba et al, (2023)a | Yes | Yes | Yes | Yes | Yes |
| Diriba et al, (2023)b | Yes | Yes | Yes | Yes | Yes |
| Diriba et al, (2024) | Yes | Yes | Yes | Yes | Yes |
| Fayehun et al, (2018) | Yes | Yes | No | No | Yes |
| Muchiri et al, (2016) | Yes | Yes | Yes | Yes | Yes |
| Saw et al, (2016) | Can’t tell | Yes | Yes | No | Yes |
| Non-randomized controlled trials | 3.1 | 3.2 | 3.3 | 3.4 | 3.5 |
| Jackson et al, (2021) | Yes | Yes | Yes | Yes | No |
| Ogunlana et al, (2018) | Yes | Yes | No | No | Yes |
| Vertue (2016) | Yes | Yes | Can’t tell | No | Yes |
| Mixed methods | 5.1 | 5.2 | 5.3 | 5.4 | 5.5 |
| Doumbia et al, (2024) | Yes | Yes | Can’t tell | Yes | No |
| Ikolaba et al, (2023) | Yes | Yes | Yes | No | Yes |

Footnote: *The numbers in the MMAT criteria column headings correspond to the numbered quality criteria in the tool according to the different study designs. This tool has yes/no/can’t tell as response options for each criterion.
